# The Formation of Neochromosomes during Experimental Evolution in the Yeast *Saccharomyces cerevisiae*

**DOI:** 10.3390/genes12111678

**Published:** 2021-10-23

**Authors:** Agnès Thierry, Varun Khanna, Bernard Dujon

**Affiliations:** Department of Genomes and Genetics, Institut Pasteur, CNRS UMR3525, F75724 Paris, France; athierry@pasteur.fr (A.T.); khanna.varun.07@gmail.com (V.K.)

**Keywords:** yeast, genome, amplification, palindrome, retrotransposon

## Abstract

Novel, large-scale structural mutations were previously discovered during the cultivation of engineered *Saccharomyces cerevisiae* strains in which essential tRNA synthetase genes were replaced by their orthologs from the distantly related yeast *Yarrowia lipolytica*. Among those were internal segmental amplifications forming giant chromosomes as well as complex segmental rearrangements associated with massive amplifications at an unselected short locus. The formation of such novel structures, whose stability is high enough to propagate over multiple generations, involved short repeated sequences dispersed in the genome (as expected), but also novel junctions between unrelated sequences likely triggered by accidental template switching within replication forks. Using the same evolutionary protocol, we now describe yet another type of major structural mutation in the yeast genome, the formation of neochromosomes, with functional centromeres and telomeres, made of extra copies of very long chromosomal segments ligated together in novel arrangements. The novel junctions occurred between short repeated sequences dispersed in the genome. They first resulted in the formation of an instable neochromosome present in a single copy in the diploid cells, followed by its replacement by a shorter, partially palindromic neochromosome present in two copies, whose stability eventually increased the chromosome number of the diploid strains harboring it.

## 1. Introduction

The total number of chromosomes forming the nuclear genome of eukaryotes often differs between related species. For example, in addition to the two sexual chromosomes (X and Y), the human genome contains 22 pairs of autosomes compared to 23 pairs for the genome of chimpanzee (*Pan troglodytes*) due to a telomere-to-telomere fusion between two ancestral ape chromosomes at the origin of chromosome 2 of modern humans [1,2]. Similarly, in the budding yeasts of the Saccharomycetaceae family, the haploid number of chromosomes varies between 6 for *Kluyveromyces lactis* and 8 for *Lachancea thermotolerans*, two species of the KLE protoploïd clade [3] or between 10 for *Nakaseomyces castelli* and 16 for *Saccharomyces cerevisiae*, two species of the post genome duplication clade [4]. By comparing yeast genome sequences, it was shown that, during evolution, the chromosome number decreases principally by telomere-to-telomere fusion between two chromosomes with the concomitant disappearance of one centromere [5]. Alternatively, the breakage of one chromosome at its centromere accompanied by the fusion of its two arms to the telomeres of other chromosomes has also been observed. By contrast, no precise mechanism has been identified yet to increase chromosome numbers, if one excepts whole-genome duplication.

In addition to chromosome number variation, eukaryotic genome evolution proceeds by numerous chromosomal rearrangements, including large scale inversions and translocations prone to gene disruption at breakpoints [6,7]. The resulting gene loss is numerically compensated by gene duplications, horizontal acquisitions and de novo gene formation, as exemplified by the reconstruction of the chromosomal architecture dynamics during the evolution of ten *Lachancea* yeast species [8], reviewed in [9]. Besides the formation of local tandem repeats, gene duplication essentially results from the segmental duplication mechanism that leads to a copy number variation of chromosomal segments between genomes [10]. Although generally limited to low-order amplifications (typically duplications), large chromosomal segments can also be multiply amplified in tandems within chromosomes, significantly enlarging their size, as recently demonstrated by experimental evolution in *S. cerevisiae* strains [11]. In these cases, some novel junctions were formed by classical exchanges between diverged sequence repeats dispersed in the yeast genome (solo LTR of ancient Ty elements). However, more remarkably, others were also formed in the absence of any sequence similarity as if they resulted from template switching within replication bubbles under stress conditions. The combination of these two mechanisms also explains the formation of multiply rearranged novel chromosomes in which low-order amplifications of large chromosomal segments are associated with extremely high amplifications of a specific gene in absence of direct selection [12].

Using the experimental evolutionary set up previously described [11], we now show the recurrent formation of neochromosomes during the evolution of diploid *S. cerevisiae* strains in which both copies of the *KRS1* gene (*YDR037w*) encoding the class II lysine-tRNA synthetase have been replaced by their ortholog (*YAL0F16291g*) from the very distantly related yeast *Y. lipolytica*. This non-reversible genetic construct confers a severely unfit phenotype to the cells (slow growth in rich medium) due to an insufficient charge of the cognate tRNA molecules. This disabled phenotype can be partially restored by a moderate increase of the number of copies of the *YAL0F16291g* gene, obtained here by the formation of entire new chromosomes eventually leading to novel lineages of *S. cerevisiae* harboring an extended diploid set.

## 2. Materials and Methods

### 2.1. Yeast Strain Origin and Nomenclature

Strains BYAT290 and BYAT581 were previously described [11]. Strain BYAT581, at the origin of all experiments described in this work, is a diploid *S. cerevisiae* of genotype *MATa/MATα*, *ura3*Δ*0/ura3*Δ*0*, *leu2*Δ*0/leu2*Δ*0*, *his3*Δ*1/his3*Δ*1*, *lys2*Δ*0/+*, *met15*Δ*0/+*, *YDR037w*::*YALI0F16291g*-*KanMX/YDR037w*::*YALI0F16291g*-*KanMX.* It has a severely unfit phenotype (doubling time of 439 min on rich YPglu medium) due to the specific replacement of its two *KRS1* (*YDR037w*) genes by their orthologs *YAL0F16291g* from *Y. lipolytica* [11].

### 2.2. Evolutionary Experiments

**Figure 1:**
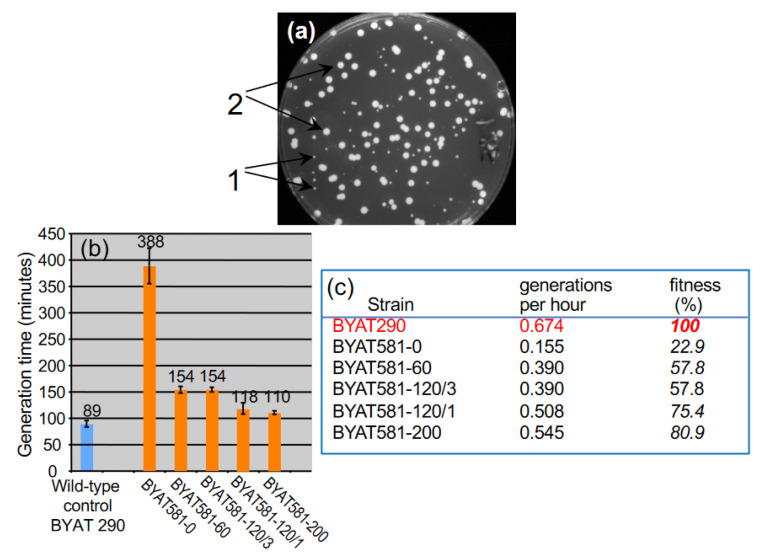
**Isolation and growth characteristics of evolved mutants.** (**a**): apparition of evolved mutants during the evolutionary trajectory of BYAT581 (Culture n°8, see Table 1). Note the clearcut difference between slow-growing colonies (1) similar to the initial BYAT581 strain, and faster-growing colonies (2) corresponding to the evolved mutants. (**b**): Colored bars: average generation times in minutes for each strain (see Section 2.3). Error bars represent 99% confidence limits (±2.6 σ/√(n − 1), where n is the number of subclones studied for each strain). (**c**): fitness expressed in % of the number of generations per hour of each strain relative to the wild-type strain BYAT290.

Strain BYAT581 was cultivated at 30 °C by serial transfers with limited population bottlenecks (10^9^ cells) in 2 liters of liquid YPD medium, as previously described [11]. The evolutionary trajectory of the culture is summarized by Table 1. At the end of each culture, an aliquot was diluted and plated on YPD medium to form single colonies, some of which were picked and studied as representative subclones of the culture after a given total number of generations. Note that, with this protocol, subclones of a given culture are only samples of the entire evolving population. They are not the direct progeny of the subclones isolated from previous cultures. We observed that the population of slow growing colonies corresponding to the unfit BYAT581 phenotype, which was visible in the first cultures, was gradually replaced in the successive cultures by faster growing colonies (Figure 1a). A slow growing colony was picked up from culture number 1 and thereafter used as control of non-evolved strain under the designation BYAT581-0. Faster growing colonies were picked up from subsequent cultures and designated “evolved mutants”. Those analyzed in this work are listed in Table 1. Evolved mutants BYAT581-120/1, BYAT581-120/3, and BYAT581-200 were themselves subsequently cultivated for a total of 145 additional generations using identical serial transfers (17 successive cultures) and subclones were isolated for molecular analysis or full genome sequencing (see Section 3.5).

### 2.3. Measurement of Generation Time of Original Construct and Evolved Mutants

Generation times of yeast strains were determined by optical density measurement in 150 μL liquid YPD cultures in paraffin-sealed 96-well microtitre plates using Sunrise TM microplate reader (TECAN) with rotational shaking. Each well was inoculated with ca. 10^4^ freshly grown cells and cultures were incubated at 30 °C with automated optical density measurements at 620 nm every 10 min for 30–50 h. Average generation times (expressed in minutes) were calculated from optical density values. For each strain, 4–16 subclones were analyzed in parallel to estimate averages and deviations.

### 2.4. Genome Sequencing and Analysis

Total DNA used for sequencing and other molecular analysis was purified from YPD grown cells of each strain as previously described [11]. Sequences were performed on Illumina equipment with average sequencing depths of 31 X–35 X for sequences read on ancient GAII machines (BYAT581-0, BYAT581-120/1, and BYAT580-200) or 170 X–392 X for sequences read on HiSeq machines (BYAT581-60, BYAT581-120/3, BYAT581-265A, BYAT581-265B, and BYAT581-345). Sequences were analyzed as described before [11,12]. Validated and trimmed reads were mapped along the reference genome sequence of S288c to measure local sequence coverage and identify possible point mutations.

### 2.5. Other Molecular Methods

PCR amplifications were done with the Taq DNA polymerase on 200 ng of yeast DNA in a 10 mM Tris-Cl buffer at pH 8.3 containing 50 mM KCl, 1.5 mM MgCl_2_, 0.01 % gelatine, and 2 mM of each desoxynucleotie triphosphate and micromolar amounts of primers.

Regular electrophoreses of yeast DNA were performed in 1% agarose gels in 1× Tris-Borate-EDTA buffer at pH 8.3 in the presence of 0.1 μg/mL of ethidium bromide. Intact chromosomal DNA used for pulsed-field gel electrophoresis was prepared from agarose-embedded yeast cells as described before [13]. Pulsed-field gel electrophoreses were run in 1% agarose gels, 0.25× Tris borate EDTA buffer pH 8.3 at 12 °C and 5 V cm*^−^*^1^ for 65 h on Rotaphor (Biometra) with an alternating field angle of 120 degrees and various pulse ramps as indicated on figures. Gel electrophoresis and transfers of enzyme-digested yeast DNA were performed according to standard methods.

## 3. Results

### 3.1. Isolation and Preliminary Characterization of Evolved Mutants

The rationale of the experiment was to let the BYAT581 population evolve spontaneously during successive cultures with bottlenecks limited to minimums of 10^9^ cells as indicated in Section 2, and to examine the evolutionary trajectory of the cell population by the analysis of subclones isolated from each culture as indicated in Table 1. BYAT581-0, showing the same unfit phenotype as BYAT581, was isolated at the start of the experiment for reference. It is not evolved. Other subclones corresponding to faster-growing colonies (as illustrated by Figure 1a) are “evolved mutants” that appeared during the successive cultures starting at number 6 (Table 1). The growth rates of BYAT581-0 and evolved mutants BYAT581-60, BYAT581-120/3, BYAT581-120/1, and BYAT581-200 were quantitatively measured (Figure 1b). Compared to the isogenic wild-type control BYAT290 bearing two copies of the *S. cerevisiae KRS1 (YDR037w)* gene, they show increased generation times indicative of their reduced fitness in rich culture medium. The generation time of BYAT581-0 is more than four times longer than that of the wild-type, consistent with the fact that the Y. lipolitica gene YALI0F16291g is a poor orthologous replacement of the *S. cerevisiae KRS1* gene resulting in an insufficient pool of lysine-charged tRNA molecules in yeast cells [11]. Compared to BYAT581-0, evolved mutants show partially restored fitness with generation times accelerated 2.5 times (BYAT581-60 and BYAT581-120/3) or 3.3–3.5 times (BYAT581-120/1 and BYAT581-200). In order to identify which mutations were able to restore the growth fitness defect caused by the irreversible genetic construct (orthologous gene replacement), the genomes of these evolved mutants were entirely sequenced and submitted to additional molecular analyses.

### 3.2. Nested Amplicons Identified from Whole Genome Sequencing of Evolved Mutants

**Figure 2:**
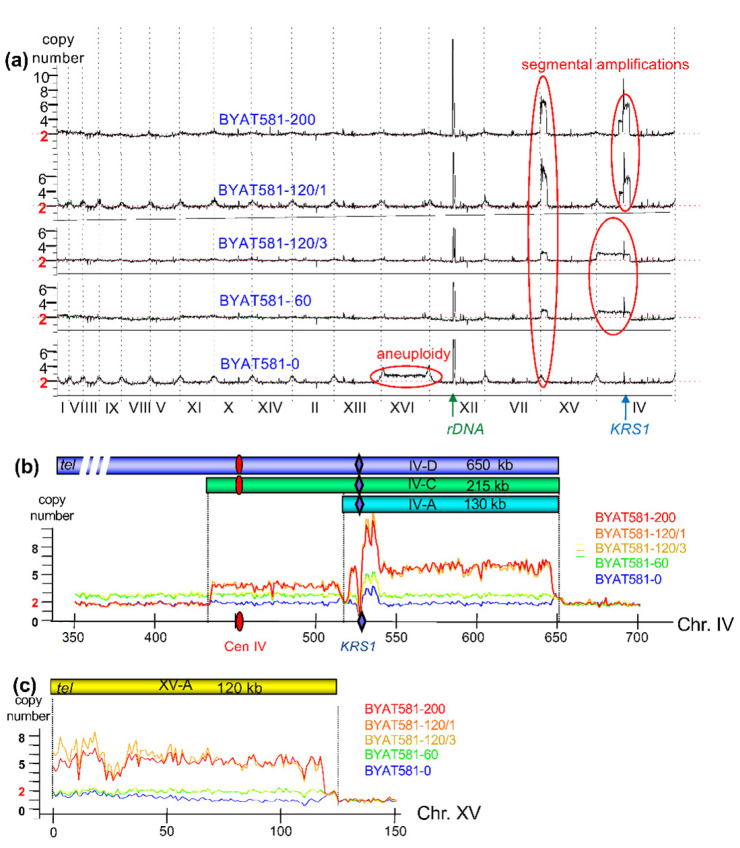
**Copy number variation along chromosomes in evolved mutants.** (**a**): For each sequenced strain, the copy number of each locus (ordinate) was plotted along the 16 chromosomes of the S288c reference sequence ranked by increasing size (abscissa, roman numerals). Copy numbers were computed from local sequence coverage (number of reads) relative to the median coverage for that strain (see Section 2.4) and normalized to 2 (diploids). Curves were smoothed using 5000 nucleotide sliding windows. The peak corresponding to rDNA was artificially cut for drawing clarity. Regions of increased copy number are red-circled. (**b**): Zoom of copy number variations along chromosome IV as determined from sequence coverage of BYAT581-0 and evolved mutants (color coded). The locations of centromere (red oval) and *KRS1* (blue diamond) are indicated. The excess sequence coverage immediately at the right of *KRS1* corresponds to the tandem copies of *ENA* genes (9 in these strains instead of 3 [11]). Horizontal bars on top materialize amplicon units. (**c**): Zoom of copy number variations along chromosome XV (same legend as in **b**).

Analysis of global sequence coverages show significant variations in two specific regions of the yeast genome, suggesting segmental amplifications in the evolved mutants (Figure 2a). The first one, visible on the left end of chromosome XV, is moderately amplified in BYAT581-60 and BYAT581-120/3 and more significantly amplified (up to six copies) in BYAT581-120/1 and BYAT581-200. The second one covers a large segment of chromosome IV in BYAT581-60 and BYAT581-120/3 and a shorter one with higher copy numbers in BYAT581-120/1 and BYAT581-200. Note the aneuploidy of chromosome XVI in BYAT581-0 (3 copies instead of 2). This aneuploidy was not observed in any of the other subclones studied and must, therefore, be regarded as accidental. It is not found in the evolved mutants because they are not the progeny of BYAT581-0 (see Section 2). No other segmental amplification could be detected in other parts of the genomes for the five strains sequenced.

A closer examination of the two regions of segmental amplifications on chromosome IV reveals a complex pattern with a moderate amplification from the left telomere to coordinate ca. 650 kb for evolved mutants BYAT581-60 and BYAT581-120/3, compared to higher amplifications between coordinates ca. 435 kb to ca. 650 kb for evolved mutants BYAT581-120/1 and BYAT581-200 (Figure 2b). Note the two distinct values at the left and right of coordinate ca. 520 kb in these two last evolved mutants. Further, note the drop in sequencing coverage at the *KRS1* locus (due to the replacement of this gene by *YALI0F16291g* in the sequenced strains) and the fluctuating and artificially high coverages immediately right to it (due to the presence of several tandem copies of the *ENA* gene) as already observed before [11]. All evolved mutants show amplification of the same segment of chromosome XV from the left telomere to coordinate ca. 120kb with, again, higher intensities for BYAT581-120/1 and BYAT581-200 than for BYAT581-60 and BYAT581-120/3 (Figure 2c).

From these data, one can postulate the existence of three partially overlapping amplicons along chromosome IV (Figure 2b) and one on chromosome XV (Figure 2c). The three chromosome IV amplicons overlap the *KRS1* locus and share the same right end. Two of them also overlap the centromere.

The longest amplicon, IV-D, extends over ca. 650 kb from the left telomere and is present in one extra copy in BYAT581-60 and BYAT581-120/3. The second longest, IV-C, extends over ca. 215 kb from the left arm (around coordinate 435 kb) and is present in two extra copies in BYAT581-120/1 and BYAT581-200. The shortest one, IV-A, extending from coordinate ca. 520 kb to ca. 650 kb within the right arm, is also present in two extra copies in BYAT581-120/1 and BYAT581-200 (bringing the corresponding segment to four extra copies considering its overlap with amplicon IV-C). The exact numbers of extra copies of amplicons in each yeast cell could be precisely calculated from sequencing coverages along chromosomal segments selected to ignore repeated sequences (such as LTR, Ty elements, and subtelomeric regions) as shown in Table 2.

No extra copies are observed in any of the six chosen segments for BYAT581-0 (calculated copy numbers range from 1.95 to 1.97, very close to 2 expected for a diploid). The same is true for the rightmost segments of chromosomes IV (coordinates from 653,607 to 1,517,675) and XV (coordinates from 124,001 to 1,081,589) for all strains (calculated copy numbers range from 1.89 to 1.98). By contrast, numbers range from 3.09 to 3.14 for the leftmost segment of chromosome XV and from 2.94 to 2.95 for the three leftmost segments of chromosome IV for strains BYAT581-60 and BYAT581-120/3 indicative of one extra copy of each of these segments in addition to the diploid number (figures are very close to 3). For strains BYAT581-120/1 and BYAT581-200, the calculated copy numbers are close to 4 (3.83 and 3.84) for the second segment of chromosome IV (indicative of two extra copies) and close to 6 (5.77 and 5.97) for the third segment of chromosome IV and for the left segment of chromosome XV (6.33 and 6.52), indicative of 4 extra copies. The three other segments are not amplified in these two strains (figures range from 1.94 to 1.99).

Merging the above figures with the map of amplicons defined in Figure 2b,c, one can conclude the presence of one extra copy of both amplicons IV-D and XV-A in strains BYAT581-60 and BYAT581-120/3, and the presence of two extra copies of both amplicons IV-C and IV-A (partially overlapping) and four extra copies of amplicon XV-A in strains BYAT581-120/1 and BYAT581-200. Pulsed-field gel electrophoreses and PCR amplifications at novel junctions were needed to determine the chromosome structures bearing these amplifications.

### 3.3. Structure of Chromosomes in Evolved Mutants

**Figure 3:**
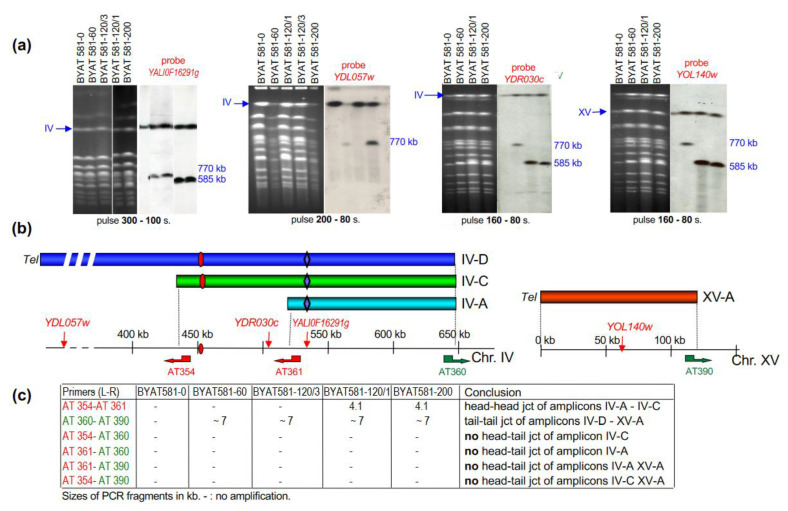
**Chromosome structures of evolved mutants as determined by PFGE and PCR amplifications at amplicon junctions.** (**a**): Results of pulsed-field gel electrophoreses hybridized with indicated genes as probes. Note the different order of samples in the different panels. (**b**): Schematic locations of probes (thin vertical arrows) and primers (thick horizontal arrows pointing leftward or rightward to indicate orientation) along chromosomal regions of amplicons (horizontal color bars). Sequences, orientations and positions of primers are indicated in Supplement Table S1. (**c**): Results of PCR amplifications using indicated primers on total DNA from indicated strains. Sizes of amplified fragments are indicated in kb.—no amplification.

Pulsed-field gel electrophoreses reveal the existence of novel bands in addition to the normal-size chromosomes IV and XV in all four evolved mutants but not in BYAT581-0 (Figure 3a). A band migrating at ca. 770 kb is visible in BYAT581-60 and BYAT581-120/3 and a shorter band migrating at ca. 585 kb is visible in BYAT581-120/1 and BYAT581-200. Both bands hybridize with the *Y. lipolytica* gene (*YALI0F16291g*) replacing *KRS1* of *S. cerevisiae* and with *YOL140w* marking the left arm of chromosome XV (BYAT581-120/3 not shown).

The 770 kb band hybridizes with probes YDL057w marking the left end of chromosome IV (BYAT581-60 is weak but visible) and YDR030c marking the right arm of chromosome IV (BYAT581-120/3 not shown). The YDR030c locus is between the left ends of amplicons IV-C and IV-A (Figure 3b). Together with its size (ca. 770 kb), these hybridizations strongly suggest that this band corresponds to the junction between extra-copies of amplicons IV-D (ca. 650 kb) and XV-A (ca. 120 kb). The formation of a 7-kb amplified fragment using primers AT360 and AT390 for PCR amplification on DNA from strains BYAT581-60 and BYAT581-120/3 (Figure 3c) confirms a tail to tail junction (hereafter designated junction n°1) of amplicons IV-D and XV-A to form a 770 kb neochromosome with one telomere at each end in these two evolved mutants. Judged from copy number measurements (Table 2), this neochromosome which bears a copy of the chromosome IV centromere (brought by amplicon IV-D) is present in a single copy per cell in otherwise diploid cells.

The 770 kb neochromosome is absent from evolved strains BYAT581-120/1 and BYAT581-200 where it is replaced by the band migrating at ca. 585 kb showing brighter hybridizations with probes *YDR030c, YOL140W*, and *YALI0F16291g* but no hybridization with the probe YDL057w. The presence of a 4.1 kb amplified fragment using primers AT354 and AT361 for PCR amplification on DNA from strains BYAT581-120/1 and BYAT581-200 suggests the presence of an inverted junction (junction n°2) between amplicons IV-C and IV-A (Figure 3c). The fact that the 7-kb fragment mentioned above is also present when these DNA are amplified using primers AT360 and AT390 indicates the presence of the junction n°1 in these strains, consistent with the fact that the right ends of amplicons IV-C and IV-A are common with amplicon IV-D. Taken together, these results suggest a partially palindromic neochromosome made of two copies of amplicon XV-A in inverted orientation, linked respectively with amplicon IV-C and IV-A (junctions n°1), themselves linked together in inverted orientation (junction n°2). The size of this neochromosome (585 kb band) fits perfectly with the cumulated sizes of amplicons XV-A (2 copies of 120 kb), IV-C (215 kb), and IV-A (130 kb). This partially palindromic neochromosome has one telomere at each end (originating from the left telomere of chromosome XV) and one centromere in its center (originating from chromosome IV and present in amplicon IV-C but not IV-A). From copy number calculation (Table 2), this neochromosome is present in two copies per cell in evolved mutants BYAT581-120/1 and BYAT581-200. This, therefore, completes the diploid chromosome set by one novel unit.

The successive appearance of evolved mutants bearing the two different neochromosomes during the evolutionary trajectory (Table 1) raises the question of their relationship. The fact that amplicons IV-A and IV-C are parts of amplicon IV-D and conserve the same junction (n°1) with amplicon XV-A suggests that the 585 kb partially palindromic neochromosome derives from the former 770 kb neochromosome. The complete collection of all evolved mutants isolated (see Table 1) can be used to examine this possibility.

### 3.4. Analysis of Other Evolved Mutants from the BYAT581 Evolutionay Trajectory

**Figure 4:**
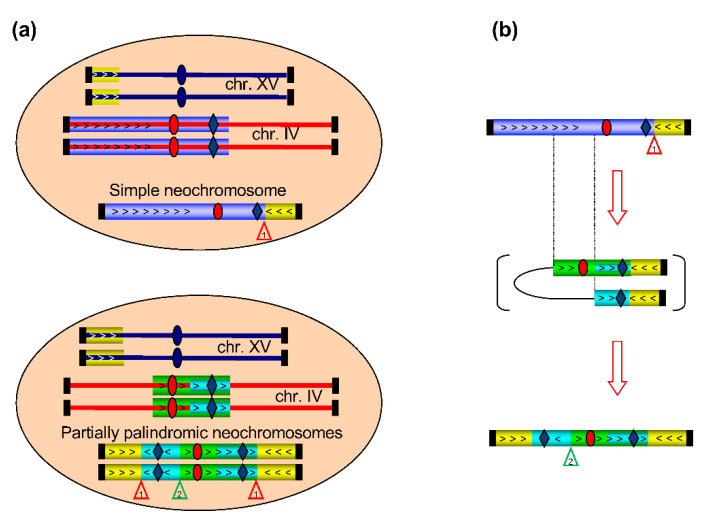
**Cartoon representation of neochromosomes in evolved mutants.** (**a**): Schematic representation of chromosomes IV and XV (red and blue lines, respectively) with centromeres (ovals), telomeres (vertical thick black bars) and neochromosomes. Amplicons are highlighted by colored bars with arrows to visualize orientation. The *KRS1* locus is symbolized by blue diamonds. **Top:** structure observed in evolved mutants BYAT581-60 and BYAT581-120/3. **Bottom:** structure observed in evolved mutants BYAT581-120/1, BYAT581-200, BYAT581- 265A, BYAT581-265B and BYAT581-345. Numbered triangles point to novel junctions formed in the neochromosomes (see text). (**b**): proposed mechanism of evolution from the simple neochromosome present in BYAT581-120/3 to the partially palindromic one present in its progeny after 145 generations (BYAT581-265A and BYAT581-265B).

DNA from these mutants was purified and analyzed by -i- PFGE probed with *YALI0F16291g* under similar conditions to Figure 3 (gel not shown) and -ii- genomic blots of XhoI and BamHI digests probed respectively with *YALI0F16291g* and *YDR098*c. Results are summarized by Table 3. It can be seen that all evolved mutants isolated from the first ten cultures (until GEN. 100) are indistinguishable from BYAT581-60 by the experiments performed, i.e., must harbor the 770 kb neochromosome made of the junction n°1 between amplicons IV-D and XV-A. This structure is still present at GEN. 110 and 120 (BYAT581-111 and BYAT581-120/3, respectively) but is not found in subsequent cultures (the five later mutants showing a 770 kb band in PFGE also show an additional band larger than 1500 kb whose nature has not been elucidated). From these results, it looks as if a first 770 kb neochromosome appeared before GEN 60 (BYAT581-60) and remained in the population until GEN 120 at which time novel structures started to appear. The first evolved mutants showing the 585 kb neochromosome is BYAT581-120/1 and this structure is found in subsequent cultures (BYAT581-130, BYAT581-140, BYAT581-150, BYAT581-190, BYAT581-200, and, probably, BYAT581-170, BYAT581-171, and BYAT581-191). A late replacement of the 770 kb neochromosome by the 585 kb neochromosome is consistent with the increased fitness of the mutants BYAT581-120/1 and BYAT581-200 compared to BYAT581-60 and BYAT581-120/3 (Figure 1c), the two latter mutants bearing six copies of the *YALI0F16291g* gene compared to three for the two former ones as schematized by Figure 4a.

### 3.5. Stability of Evolved Mutants in Subsequent Evolutionary Experiments

The stability of the different evolved mutants offers another criterion to examine their possible relationship. Evolved mutants BYAT581-120/3 and BYAT581-200 harboring, respectively, the 770 kb neochromosome and the 585 kb partially palindromic neochromosome were, therefore, grown separately for additional generations in YPD medium using the same serial transfer method. A total of 17 successive cultures were made and one subclone of each culture was isolated after ca. 35, 70, 110, and 145 generations (cultures n°4, 8, 13, and 17, respectively) and submitted to PFGE analysis as before. Results show complete stability for the 585 kb partially palindromic neochromosome (all four subclones show the 585 kb band; Table 4). By contrast, only the first two subclones of BYAT581-120/3 show the 770 kb band. The last subclone isolated after 145 generations shows a complex pattern similar to what was previously observed with evolved mutant BYAT581-201 (see Table 3).

Three other subclones were also isolated after 145 generations and their genomes were fully sequenced. Subclone BYAT581-345 shows the same amplification pattern as BYAT581-200 from which it derives after 150 additional generations. This pattern corresponds to the 585 kb neochromosome, confirming the stability of this structure (Supplementary Figure S1). However, more surprisingly, the two subclones, BYAT581-265A and BYAT581-265B, do not show the same amplification pattern as BYAT581-120/3 bearing the 770 kb neochromosome from which they derive. Instead, they exhibit the amplification pattern typical of the 585 kb neochromosome. Thus, we can conclude that the original neochromosome of 770 kb is an unstable structure that eventually gives rise to the 585 kb partially palindromic neochromosome. The common parts between these two neochromosomes suggests a simple mechanism for their successive formation, as illustrated by Figure 4b. However, a more detailed examination of the junctions is needed before a final conclusion can be reached.

### 3.6. Extremities of Amplicons and Molecular Structures of Junctions

The ends of amplicons can be precisely mapped to the yeast genome sequence from the abrupt variation of sequence coverage along chromosomes, provided that the global sequencing depth of evolved mutants is sufficient (Supplementary Figure S2). On chromosome IV, the excess sequence coverage clearly visible in BYAT581-265A, BYAT581-265B, and BYAT581-345 defines the common right end of amplicons IV-D and IV-C close to coordinate 645,500. The right end of amplicon IV-A is less clear on the patterns of BYAT581-60 and BYAT581-120/3 due to its lower copy number but is consistent with the same coordinates between 645,500 and 650,000. Using the same analysis, the left ends of amplicons IV-C and IV-A present in BYAT581-265A, BYAT581-265B, and BYAT581-345 appear, respectively, close to coordinates 434,800 and 519,800. Note that amplicon IV-D present in BYAT581-60 and BYAT581-120/3 reaches the left telomere. The same method applied to chromosome XV locates the right end of amplicon XV-A between coordinates 117,000 and 124,000.

**Figure 5:**
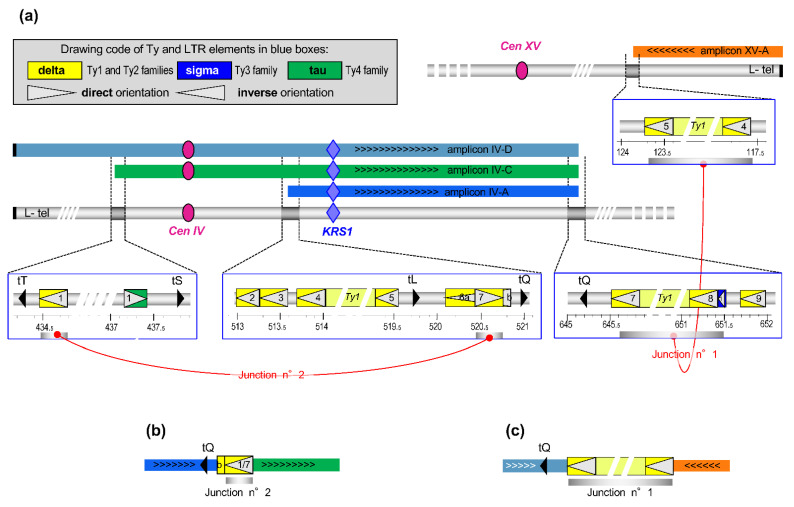
**Junctions between amplicons.** (**a**): Schematic representation of parts of chromosome IV and XV (silver horizontal bars) with amplicons (colored bars) and zooms on regions of amplicon ends (blue boxes) showing the presence of Ty elements, LTRs (drawing code in top insert) and tRNA genes (black triangles). Note the inverted orientation of chromosome XV to facilitate drawing. Elements involved in junctions are highlighted by shadowed bars joined by red lines. (**b**): junction n°2 between the left ends of amplicons IV-C and IV-A (inverted) occurred within LTR1 and LTR7. (**c**): junction n°1 between the right end of amplicon XV-A (inverted) and the right end common to amplicons IV-D, IV-C and IV-A occurred within the Ty 1 element.

The sequences of chromosomes IV and XV reveal the presence of Ty elements and/or numerous solo LTRs at above coordinates (Figure 5a). Considering the orientations and locations of primers AT354 and AT361 on chromosome IV (see Figure 3c) and the fact that the PCR fragment they amplify on BYAT581-120/1 and BYAT581-200 DNAs measures 4.1 kb, it appears that the inverted junction between amplicons IV-C and IV-A must involve a recombination between LTR1 and LTR7 (Figure 5b). These two delta elements are in inverted orientation on chromosome IV (explaining the inverted junction) and share an average of only 63% nucleotide identity over their 317 bp common length. Their longest segment of identical sequence extends over only 27 nucleotides in which the exchange is likely to have taken place in junction n°2.

The presence of a full length Ty1 element with its two LTRs (YDRCTy1-1) between coordinates ca. 645.5 and 651.4 kb of chromosome IV and of another one (YOLWTy1-1) between coordinates ca. 117.7 and 123.6 kb of chromosome XV strongly suggests that a recombination occurred between them to form the junction between amplicon XV-A and the right ends of amplicons IV-D, IV-C, and IV-A (Figure 5c). The orientation of these two Ty elements explains the formation of a neochromosome with two telomeres. Such a recombination is consistent with the formation of the ca. 7 kb fragment when primers AT360 and AT390 are used for PCR amplification on DNA from evolved mutants (Figure 3c), but the exact point of exchange cannot be more precisely mapped because the two Ty elements share an average of 95% nucleotide identity over their 5926 common length.

## 4. Discussion

Chromosomal rearrangements occur frequently in eukaryotic cells and produce apparently unlimited varieties of normal or pathological results, prone to act in speciation, evolution, or diseases such as cancers [14,15,16,17,18]. Here, we show that they also allow the rapid phenotypic recovery from non-reversible deleterious mutations artificially created in the yeast *S. cerevisiae*. This conclusion is not novel. Using the same experimental set up, we have previously shown rapid phenotypic restorations originating from high-order segmental amplifications within chromosomes [11] or complex intrachromosomal rearrangements associated with the massive amplification of a non-selected gene [12]. The novelty here relies on the fact that new chromosomes with one centromere and two telomeres are added to the yeast genome by the simple junctions between extra copies of large chromosomal segments. The first junction observed probably results from a classical event of homologous recombination between two highly similar Ty elements that happen to be located in appropriate inverted orientations on two chromosomes. Similar events have previously been reported [19]. However, the other junction involves an exchange between two LTR elements of the same chromosome, extensively diverging in sequences. Similar events, involving other LTR elements equally diverging in sequences, were also previously observed in evolutionary experiments using the same experimental set up and were interpreted as reflecting template switching within replication bubbles rather than homologous recombination [11,12]. Interestingly, this event occurred repeatedly here because the genomes of BYAT581-265A and BYAT581-265B, two subclones of BYAT581-120/3 in which this junction was not present, are not distinguishable from those of BYAT581-120/1 and BYAT581-200 which appeared during the main evolutionary trajectory of BYAT581.

The subsequent experimental evolution of BYAT581/3 reported here (Table 4 and Supplementary Figure S1) directly demonstrates that the 770 kb neochromosome, present in one copy per cell, eventually gives rise to the 585 kb partially palindromic neochromosome, present in two copies per cell, as was also suggested by the successive appearance of evolved mutants in the original evolutionary experiment (Table 3). This evolution, which requires the formation of a novel junction (see Figure 4), is consistent with the fact that the evolved mutants bearing the 585 kb partially palindromic neochromosome have a slightly better growth rate than those bearing the 770 kb neochromosome (Figure 1b), hence creating a potentially unstable situation in cultures if the 770 kb neochromosome can mutate to form the 585 kb neochromosome as shown here. It is also possible that, in diploid cells, the presence of a neochromosome in two copies per cell generates more stable mitoses compared to the presence of a neochromosome in only one copy per cell. It is unknown why the 770 kb neochromosome was only found in one copy per cell. One possibility is the existence of a gene on the left arm of chromosome IV (present in amplicon IVA but absent in amplicon IC) which creates a deleterious effect when present in excessive copy number.

It is interesting to note that the evolutionary trajectory of BYAT581 described here is entirely different from the previously described evolutionary trajectory of BYAT583, although both diploid strains, derived from meiotic products of the same ancestral diploid strain (BYAT526), have exactly the same genotype (see Supplementary Table S1 in Reference [11]) and were placed in identical experimental conditions. In the latter case, in addition to classical events of segmental duplication or circular episome formation, a high order amplification of an internally deleted internal segment of chromosome IV was observed, bringing the total number of the *YAL0F16291g* gene to more than 20 copies per cell (much more than what was needed to restore fitness) and extending the chromosome size by ca. 1Mb [11]. Here, the addition of only one extra copy of *YAL0F16291g* (BYAT581-60 and BYAT581-120/3) proves sufficient to approximately double the growth rate of the parental strain, and the addition of four copies (BYAT581-120/1 and BYAT581-200) restores the growth fitness to near normal level (Figure 1c). Therefore, if the selection pressure resulting from the orthologous replacement of the essential *KRS1* gene by *YAL0F16291g* is needed to select the chromosome rearrangements observed, those rearrangements must be driven by internal mechanisms of chromosomal dynamics rather than by the selection of their products. This is fully consistent with the massive amplification (up to 880 copies per cell) of an unselected gene previously reported [12]. The idea that gene amplification is a final consequence of internal mechanisms of chromosomal dynamics rather than their driving force is also consistent with the very different evolutionary trajectory observed in this work compared to previous results [11,12], because in each culture, the early stochastic events determine the possibilities of subsequent events, yielding diverging results. The fact that, in the present experiments, the complex formation of a neochromosome happened instead of a simple aneuploidy that would have resulted in the same number of copies of the *YAL0F16291g* gene in the cell exemplifies the multiplicity of possible routes in chromosomal dynamics. The formation of neochromosomes to accelerate the growth rate in yeast cells is obviously reminiscent of their occurrence in human cancer cells where, despite their broader diversity and distinct mechanism of formation, neochromosomes also play some role in their proliferation [20].

## Figures and Tables

**Table 1:** Evolutionary trajectory of the BYAT581 population.

Culture Number	Culture Duration (Days)	Final Cell Density /mL (×10^8^)	nb of Gen. in Culture (n)	Cumulate Number of Generations	GEN.	First Evolved Mutant Isolated	Second Evolved Mutant Isolated
**1**	**3**	**1.0**	**11.0**	**11.0**	**0**	BYAT581-0 ^(^*^)^	
2	7	0.5	7.6	18.6			
3	4	1.7	8.4	27.0			
4	2	1.5	8.2	35.2			
5	6	1.1	7.8	43.0			
6	3	1.1	7.8	50.8	60	**BYAT581-60**	BYAT581-61
7	3	1.1	7.8	58.6	70	BYAT581-70	BYAT581-71
8	2	1.1	7.8	66.4	80	BYAT581-80	BYAT581-81
9	2	1.0	7.6	74.0	90	BYAT581-90	BYAT581-91
10	3	1.0	7.6	81.7	100	BYAT580-100	BYAT580-101
11	2	1.0	7.6	89.3	110	BYAT580-110	BYAT580-111
12	2	0.8	7.3	96.6			
13	5	1.0	7.6	104.3	120	**BYAT581-120/1**	BYAT581-121
						**BYAT581-120/3**	
14	2	1.1	7.8	112.1	130	BYAT581-130	BYAT581-131
15	3	0.9	7.5	119.5	140	BYAT581-140	BYAT581-141
16	2	1.0	7.6	127.2	150	BYAT581-150	BYAT581-151
17	2	0.9	7.5	134.7			
18	3	0.9	7.5	142.2			
19	4	1.1	7.8	150.0	170	BYAT581-170	BYAT581-171
20	3	1.2	7.9	157.9			
21	4	1.0	7.6	165.5	190	BYAT581-190	BYAT581-191
22	10	1.1	7.8	173.3			
23	10	1.5	8.2	181.5	200	**BYAT581-200**	BYAT580-201

First five columns: details of the serial transfer cultures. At the end of each 2-liter culture in YPD liquid medium, the final cell density (d, column 3) was estimated by Malassez cell counting (on diluted aliquots), and the number of cellular generations in the culture (n, column 4) was deduced using the equation n = (ln (Nf) − ln (Ni))/ln (2) where Nf is the final cell number in the culture (Nf = 2000 × d) and Ni the initial cell number (10^9^ cells of the previous culture, except for cultures 1 (10^8^) and 2 (5 × 10^8^)). The cumulated number of generations since the original inoculum is indicated in column 5. Last two columns: list of evolved mutants isolated from fast growing colonies after the various cultures. (*) Note that BYAT581-0 isolated from a slow growing colony after the first culture is representative of the original unfit strain BYAT581. It is not an evolved mutant. For convenience, evolved mutants are designated by an approximate number of generations, GEN, (compare to actual numbers in column 5). Bold type highlights fully sequenced strains isolated after cultures n°1, 6, 13 and 23 (see text).

**Table 2:** Calculation of precise copy numbers per cell of selected chromosome segments.

	Chromosome IV Segment Coordinates			Chromosome XV Segment Coord.		
Strain	from 16,204 to 433,994	from 438,047 to 512,109	from 539,803 to 645,035	from 653,607 to 1,517,675	from 19,491 to 113,130	from 124,001 to 1,081,589
BYAT581-200	1.99	**3.84**	**5.97**	1.94	**6.33**	1.97
BYAT581-120/1	1.95	**3.83**	**5.77**	1.96	**6.52**	1.94
BYAT581-120/3	**2.95**	**2.95**	**2.95**	1.89	**3.09**	1.98
BYAT581-60	**2.94**	**2.94**	**2.94**	1.92	**3.14**	1.98
BYAT581-0	1.95	1.95	1.95	1.95	1.97	1.95

Total number of sequence reads per kb in indicated segments were divided by total number of sequence reads per kb on chromosome VII (not amplified) in the same strain and normalized to 2 (diploid) to obtain segmental copy numbers per cell. Figures differing significantly from 2 are bold underlined.

**Table 3:** Molecular analysis of all evolved mutants of the BYAT581 evolutionary trajectory.

Mutant N°	PFGE Probed with *YALI0F16291g*	Genomic Blots of	
		*Xho*I Digest Probed with *YALI0F16291g*	*Bam*HI Digest Probed with *YDR098c*
**BYAT581-0**	**IV**	**12**	**7**
**BYAT581-60**	**IV + 770 kb**	**12**	**7 + 12**
BYAT581-61	IV + 770 kb	-	-
BYAT581-70	IV + 770 kb	-	-
BYAT581-71	IV + 770 kb	-	-
BYAT581-80	IV + 770 kb	-	-
BYAT581-81	IV + 770 kb	-	-
BYAT581-90	IV + 770 kb	12	-
BYAT581-91	IV + 770 kb	-	-
BYAT581-100	IV + 770 kb	-	-
BYAT581-101	IV + 770 kb	-	-
BYAT581-110	IV + 770 kb (+>1500 kb)	12 (+11)	
BYAT581-111	IV + 770 kb	12	-
BYAT581-120/1	IV + 585 kb	12 + 15	-
**BYAT581-120/3**	**IV + 770 kb**	12	7 + 12
BYAT581-130	IV + 585 kb	-	-
BYAT581-131	IV + 770 kb (+>1500 kb)	12	
BYAT581-140	IV + 585 kb	-	-
BYAT581-141	IV + 770 kb (+>1500 kb)	12 (+6.5)	
BYAT581-150	IV + 585 kb	-	-
BYAT581-151	IV + 770 kb (+>1500 kb)	12	
BYAT581-170	-	12 + 15	-
BYAT581-171	-	12 + 15	-
BYAT581-190	IV + 585 kb	12 + 15	-
BYAT581-191	-	12 + 15	-
**BYAT581-200**	**IV + 585 kb**	**12 + 15**	**7 + 12**
BYAT581-201	IV + 770 kb + 585 kb (+ >1500 kb) ^(1)^	-	-

PFGE were run as indicated in Section 2.5 with pulse ramps of 300 to 100 s. The table indicates the sizes of hybridizing fragments in addition to chromosome IV. Genomic blots were performed on total DNA digested with *Xho*I or *Bam*HI and the sizes of hybridizing fragments are indicated in kb. The 12 kb B*am*HI fragment corresponds to inverted junction n°1 between amplicons IV-D and XV-A (the normal chromosome IV sequence yields the 7 kb fragment). The 15 kb *Xho*I fragment corresponds to the inverted junction n°2 between amplicons IV-C and IV-A (the normal chromosome IV sequence yields the 12 kb fragment). All hybridizations were made with gel-purified PCR products as probes. Bold types highlight sequenced strains (PFGE images are in Figure 3a). -: not done. Unexplained bands are under brackets. Note (1) another extra band of size 1400 kb is also visible on EB staining of PFGE gel for this strain.

**Table 4:** Stability of the 770 kb neochromosome and the 585 kb partially palindromic neochromosome.

Subclone Isolated after *n* Generations	PFGE Probed with *YALI0F16291g*	
	Subclones of BYAT581-120/3	Subclones of BYAT581-200
*n* = 35	IV + 770 kb	IV + 580 kb
*n* = 70	IV + 770 kb	IV + 580 kb
*n* = 110	-	IV + 580 kb
*n* = 145	IV + 770 kb +580 kb (+>1500 kb)	IV + 580 kb

Same legend as Table 3.

## Data Availability

All data are available upon request to athierry@pasteur.fr.

## Supplementary Materials

The following are available online at https://www.mdpi.com/article/10.3390/genes12111678/s1, **Table S1:** Sequence: orientation and position of primers used in this work. **Figure S1:** Copy number variations along chromosomes in late progenies of evolved mutants. **Figure S2:** Images of Illumina deep sequencing of evolved mutants mapped to the reference S288C genome sequence [21].
