# Supplementary material for: The Formation of Neochromosomes during Experimental Evolution in the Yeast Saccharomyces cerevisiae
Source: Genes (Basel). 2021 Oct 23;12(11):1678. doi: 10.3390/genes12111678 (PMC8619081; doi:10.3390/genes12111678)
Supplement: Supplementary file 1 [file genes-12-01678-s001.zip › genes-1409691-supplementary.pdf]

Suppl. Table S1: Sequence, orientation and position of primers used in this work.

Primer	Sequence (5' – 3')	Chrom.	Strand	Coord. 5' end
AT 354	GTCCAGATGATACACCTTGT	IV	minus	438,050
AT 360	GATGGAACCTGAAAAGAACAC	IV	plus	645,011
AT 361	GCATAATGCATATTTTGATGATGC	IV	minus	521,318
AT390	GAGTCATGGAATTCAGTAC	XV	plus	117,371

Figure S1: Copy number variations along chromosomes in late progenies of evolved mutants.

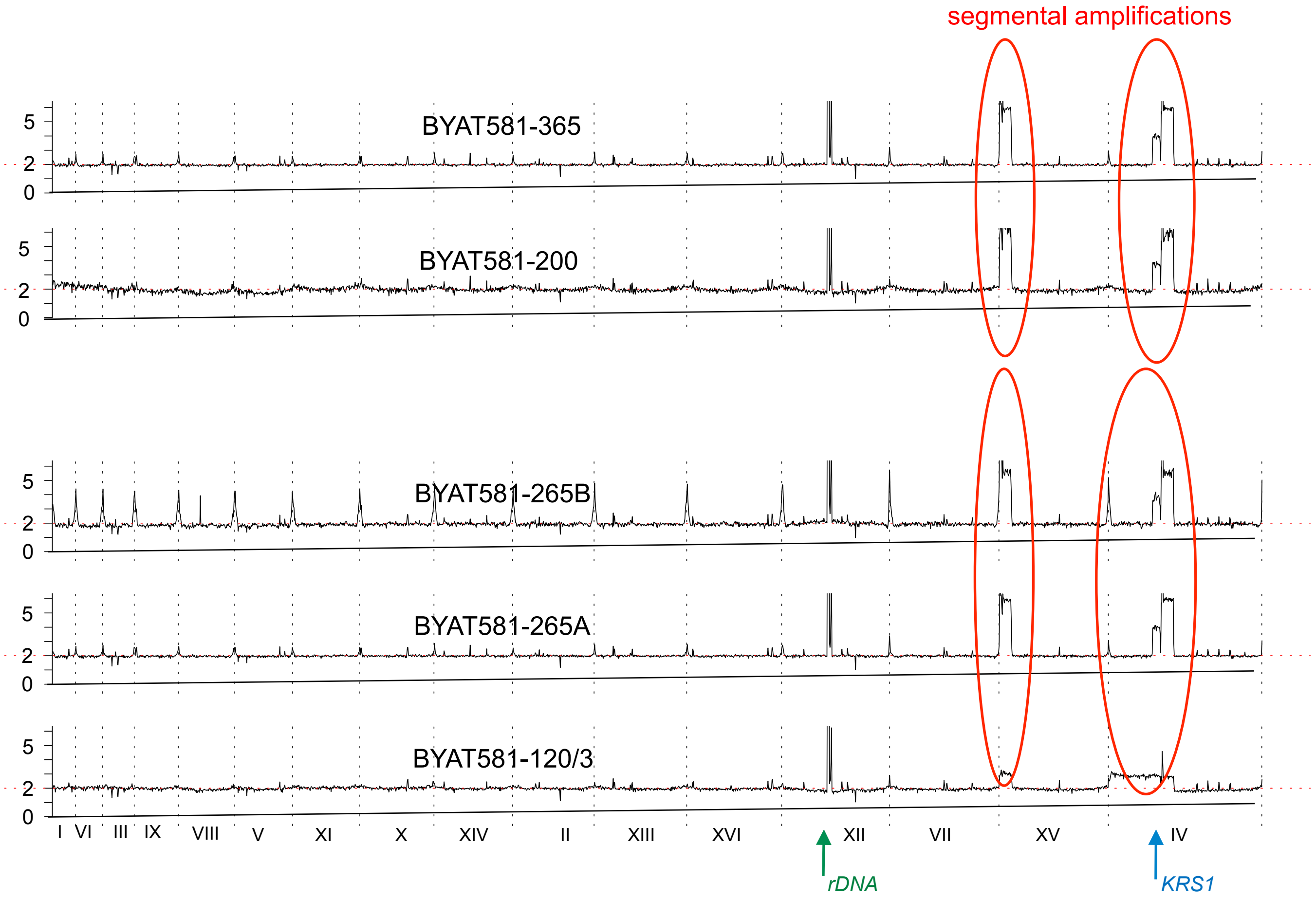

Same legend as Figure 2a. Strains BYAT581-265A and BYAT581-265B are subclones of BYAT581-120/3 after 145 generations in YPD medium. Strain BYAT581-345 is a subclone of BYAT581-200 after 145 generations in YPD medium.. Data for BYAT581-120/3 and BYAT581-200 are taken from Figure 2a.

Suppl. Figure S2: Images of Illumina deep sequencing coverages of evolved mutants mapped to the reference S288C genome sequence.

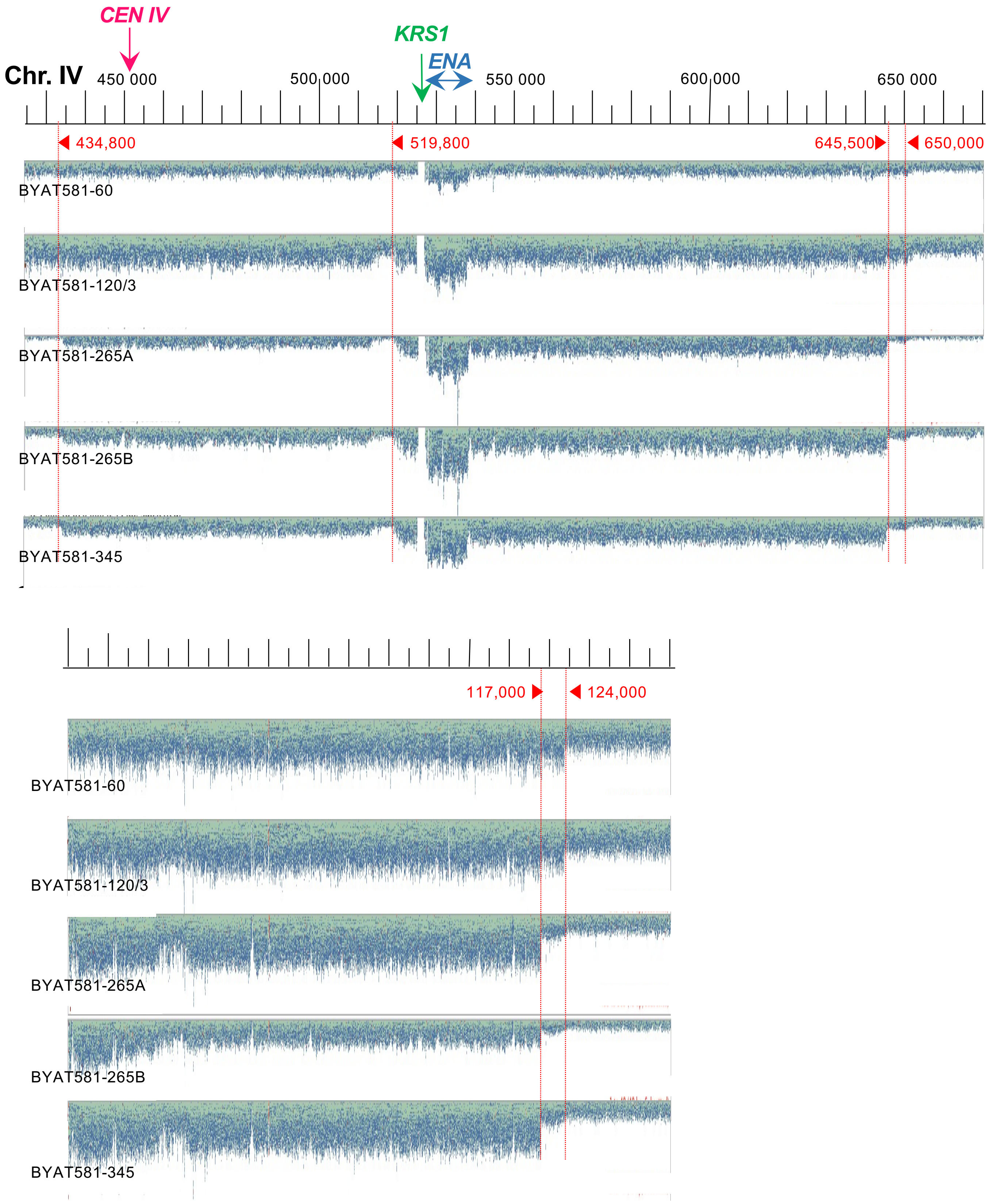

The *Tablet* [1] images of sequence coverages are shown for regions of chromosomes IV and XV corresponding to amplicons IV-C, IV-A and XV-A. Evolved mutants bearing amplicons IV-D and XV-A were sequenced at average depths of 392 X and 305 X, respectively, for BYAT581-60 and BYAT581-120/3. Evolved mutants bearing amplicons IV-C, IV-A and XV-A were sequenced at average depths of 269 X, 170 X and 290 X, respectively, for BYAT265A, BYAT582-265B and BYAT581-345. Vertical red lines locate deduced amplicon ends on the chromosome maps.

[1] Milne, I.; Bayer, M.; Stephen, G.; Cardle, L.; Marshall, D. Tablet: Visualizing Next-Generation Sequence Assemblies and Mappings. Methods Mol Biol. **2016** *1374*:253-268. doi: 10.1007/978-1-4939-3167-5_14.
